# Biological Evaluation of a New Sodium-Potassium Silico-Phosphate Glass for Bone Regeneration: In Vitro and In Vivo Studies

**DOI:** 10.3390/ma14164546

**Published:** 2021-08-13

**Authors:** Elisa Fiume, Dilshat U. Tulyaganov, Avzal Akbarov, Nigora Ziyadullaeva, Andrea Cochis, Alessandro C. Scalia, Lia Rimondini, Enrica Verné, Francesco Baino

**Affiliations:** 1 Department of Applied Science and Technology, Politecnico di Torino, 10129 Turin, Italy; elisa.fiume@polito.it (E.F.); enrica.verne@polito.it (E.V.); 2 Department of Natural-Mathematical Sciences, Turin Polytechnic University in Tashkent, Tashkent 100095, Uzbekistan; tulyaganovdilshat@gmail.com; 3 Department of Prosthodontics, Tashkent State Dental Institute, Tashkent 100047, Uzbekistan; avzal@rambler.ru (A.A.); nigorazstom@yandex.ru (N.Z.); 4 Center for Translational Research on Autoimmune and Allergic Disease—CAAD, Department of Health Sciences, 28100 Novara, Italy; andrea.cochis@med.uniupo.it (A.C.); alessandro.scalia@uniupo.it (A.C.S.); lia.rimondini@med.uniupo.it (L.R.)

**Keywords:** bioactive glass, biocompatibility, in vitro, in vivo, bone regeneration

## Abstract

In vitro and in vivo studies are fundamental steps in the characterization of new implantable materials to preliminarily assess their biological response. The present study reports the in vitro and in vivo characterizations of a novel experimental silicate bioactive glass (BG) (47.5B, 47.5SiO_2_-10Na_2_O-10K_2_O-10MgO-20CaO-2.5P_2_O_5_ mol.%). Cytocompatibility tests were performed using human mature osteoblasts (U2OS), human mesenchymal stem cells (hMSCs) and human endothelial cells (EA.hy926). The release of the early osteogenic alkaline phosphatase (ALP) marker suggested strong pro-osteogenic properties, as the amount was comparable between hMSCs cultivated onto BG surface and cells cultivated onto polystyrene control. Similarly, real-time PCR revealed that the osteogenic collagen I gene was overexpressed in cells cultivated onto BG surface without biochemical induction. Acute toxicity tests for the determination of the median lethal dose (LD_50_) allowed classifying the analyzed material as a slightly toxic substance with LD_50_ = 4522 ± 248 mg/kg. A statistically significant difference in bone formation was observed in vivo through comparing the control (untreated) group and the experimental one, proving a clear osteogenic effect induced by the implantation at the defect site. Complete resorption of 47.5B powder was observed after only 3 months in favor of newly formed tissue, thus confirming the high osteostimulatory potential of 47.5B glass.

## 1. Introduction

Bioactive glasses (BGs) are seen as attractive bone substitute materials in human medicine due to their ability to bond both to hard and to soft tissues, creating a stable interface while promoting cell viability, healthy tissue regeneration and angiogenesis [1,2,3,4,5].

The era of BGs began in 1969 with the invention of the first bioactive composition, known by the name of 45 S5 Bioglass^®^ (45SiO_2_-24.5CaO-24.5Na_2_O-6P_2_O_5_ wt.%), which was tested in vivo in a rat femoral implant model proposed by Dr. T.K. Greenlee at the Department of Orthopedics at the University of Florida. Preliminary in vitro and in vivo results were published in 1971 [6,7,8], providing an explanation for the interfacial bonding of the glass implant to bone [9]. Specifically, it was demonstrated that the bioactive mechanism relied on the ionic dissolution of glass and led to osteointegration and osteogenetic response, resulting in faster regenerative pathways.

In subsequent years, these revolutionary results strongly motivated further studies aimed at optimizing the biological response of bone tissue to the exposure to BG-based grafts and their dissolution products, developing new bioactive compositions by the introduction of specific therapeutic ions able to confer multiple therapeutic actions to the material upon implantation and reaction in contact with the physiological environment [10,11].

When developing new bioactive materials, in vitro cell culture studies represent the first significant indicator of the response of a simplified biological system in contact with the grafting material, thus constituting a key step of their characterization. These tests can provide information about cytotoxicity, genotoxicity, cell proliferation and differentiation and are used primarily as a first-stage test to avoid unnecessary use of animals in the testing of cytologically inappropriate materials [12]. Parameters used for cell culture studies have to be properly selected according to the final application (i.e., the intended use of the selected BG composition), the cell types and the specific assays to be used [13].

Among the most important indicators in a preliminary phase, cytotoxicity and cell viability are usually evaluated as they are relatively fast, low cost and reveal a promising potential for automation. Moreover, in some cases, in vitro tests on human cells can be considered significantly more representative of the biological response of a given system than experimental results coming from animal in vivo studies [12]. Despite this, animal models still represent a mandatory path towards the validation of both clinical devices and drugs. In vitro testing, in fact, is usually confined to the response of individual cell lines or primary cells and is not fully able to demonstrate the tissue response to the biomaterial [14].

Over the years, BGs with different compositions have been tested in relevant bone defect models. It was shown that the bone regenerative capacity of BGs in vivo is the result of different factors including the compositional system (e.g., silicate, borate or phosphate glasses, presence of dopants, etc.) and the synthesis route (melt-quenching or sol-gel). Moreover, when BGs are used for producing 3D porous scaffolds, the manufacturing process selected for obtaining the graft should also be evaluated, as it determines scaffold porosity and morphological features, influencing cell migration, bone tissue ingrowth and mass transport properties [15]. Apart from these relevant aspects, the selected animal model, the defect size and anatomical location, as well as the implantation period can also considerably affect the final outcome of an in vivo experiment. Usually, in preliminary tests, rats and rabbits are preferred due to their small size and ease of handling [15]. One of the major concerns related to the in vivo implantation of BG-based products is related to the potential toxicity of degradation products, which can provoke secondary side effects in vital high-metabolic-rate organs participating in the excretion of waste substances [16].

Acute toxicity is an initial procedure for general toxicological screening of chemical and pharmacological agents that aims to establish the dose-dependent adverse effect caused by single or multiple administration of the tested substance within 24 h. Traditionally, an acute toxicity study is conducted to experimentally define the dose that kills 50% of the test animal population, denoted as LD_50_. Importantly, the absolute values of LD_50_ for a compound may vary due to differences in protocol details, animal model used, caging and test-chemical source, etc. Besides LD_50_, alternative methods for testing of acute toxicity were recently proposed [17,18,19,20,21].

In this work, the biological response of an experimental sodium-potassium silico-phosphate glass named 47.5B, originally developed at Politecnico di Torino for potential use in bone regenerative strategies [22], was investigated by performing both in vitro and in vivo experiments comprising both acute toxicity and in vivo animal biocompatibility testing.

Previous studies performed on 47.5B BG revealed an exceptional bioactive behavior [23], attractive thermal properties [24] and good processability by different technological approaches [25,26,27,28,29], suggesting high potential to be used as basic material for the production of 3D porous bone substitutes with adequate mechanical properties [30,31] and enhanced hydroxyapatite-forming ability [25,26,27,28,29]. It was demonstrated, in fact, that the selected composition preserves fast apatite-conversion kinetics even in the form of a glass-ceramic material obtained upon high-temperature thermal treatments, which are usually necessary to obtain well-densified struts by sintering of glass particles [27]. Therefore, all these promising results from previous studies motivated the need for biological assessment of the material.

## 2. Materials and Methods

### 2.1. Glass Production

The silicate glass used in this work is called 47.5B (47.5SiO_2_-10Na_2_O-10K_2_O-10MgO-20CaO-2.5P_2_O_5_ mol.%) [22] and was obtained by melting a homogeneous mix of the powdered precursors (SiO_2_, Na_2_CO_3_, K_2_CO_3_, (MgCO_3_)_4_·Mg (OH)_2_·5 H_2_O, CaCO_3_ and Ca_3_(PO_4_)_2_, Sigma-Aldrich, St. Louis, MO, USA) up to 1500 °C in a platinum crucible.

For the production of bulk samples, used for cell seeding during in vitro tests, the melt was poured into a hot brass cylindrical mold with diameter of 10 mm and immediately placed inside a furnace preheated at 500 °C to perform a 10 h annealing treatment, in order to obtain a glass rod with no residual internal stresses. The annealing treatment was performed slightly below the temperature of glass transition of the material (T_g_ ≈ 530 °C), assessed in previous studies by differential thermal analysis (DTA) measurements [24]. Before being extracted, the rod was left to slowly cool down inside the furnace to prevent the risk of thermal shock. Then, 2 mm thick 47.5B glass slices were obtained by means of a tabletop precision saw (Buehler, IsoMet™ High Speed Pro, Lake Bluff, IL, USA). 

Prior to cell exposures, the surfaces of the samples were hand-polished by using 320–4000 grit SiC papers at 500 rpm. After being polished, samples were immersed in an ultrasonic bath for approximately 5 to 10 min, to remove residual debris.

For the production of BG powders to be used for in vivo tests, a glass frit was produced by quenching in distilled water; then the material was milled (Pulverisette 0, Fritsch, Idar-Oberstein, Germany) and sieved (stainless steel sieve, Giuliani Technology Srl, Turin, Italy) to a final grain size below 32 μm.

### 2.2. In Vitro Studies

#### 2.2.1. In Vitro Cytocompatibility Evaluation

Bulk 47.5B specimens were heat-sterilized at 180 °C for 1 h and stored at room temperature prior to being used for biological assessments. Polystyrene was used as substrate for the positive control group and test results were normalized towards it. Preliminary cytocompatibility of 47.5B glass was evaluated on three different cellular phenotypes, which were representative of the tissues potentially in contact with the implant material at the defect site: human mature osteoblasts (U2OS, ATCC HTB-96), human mesenchymal stem cells (hMSCs, ATCC PCS500012) and human endothelial cells (EA.hy926, ATCC CRL-2922). Cells used for experiments were purchased from the American Type Culture Collection (ATCC, Manassas, VA, USA). hMSCs were cultivated in low-glucose Dulbecco’s modified Eagle medium (DMEM, Sigma-Aldrich) supplemented with 15% fetal bovine serum (FBS, Sigma) and 1% antibiotics (penicillin/streptomycin) at 37 °C, 5% CO_2_ atmosphere. U2OS were cultured in high-glucose DMEM (Sigma-Aldrich) supplemented with 10% FBS, 1% antibiotics at 37 °C and 5% CO_2_ atmosphere. EA.hy926 were cultured in high-glucose DMEM (Sigma-Aldrich) supplemented with 10% FBS, 1% antibiotics at 37 °C and 5% CO_2_ atmosphere. Cells were cultivated up to 80–90% confluence and then detached by trypsin-EDTA solution, harvested and used for experiments.

In order to test in vitro cytocompatibility of specimens, cells were directly seeded onto specimen surface in a defined concentration (1 × 10^4^ cells/specimen) and cultivated for 1, 2 and 3 days. At each time point, the viability of the cells in direct contact with specimens was evaluated by means of the metabolic colorimetric Alamar blue assay (Alamar Blue™, Life Technologies–Thermo Fisher, Waltham, MA, USA) following the manufacturer’s instructions. Briefly, supernatant was removed from each well and replaced with the Alamar blue solution (10% *v*/*v* in fresh medium). Plates were incubated in the dark for 4 h and then 100 μL was removed, spotted into a new black 96-well plate and fluorescence signals were evaluated by a spectrophotometer (Spark, Tecan Trading AG, Mannedorf, Switzerland) using a fluorescence excitation wavelength of 570 nm and a fluorescence emission reading of 590 nm. Moreover, the adhesion, spread and morphology of cells cultivated onto the specimen surface were visually checked by digital light microscopy (Invitrogen EVOS Floid, from Thermo Scientific, Waltham, MA, USA).

#### 2.2.2. Pro-Osteogenic Efficacy

In order to verify the potential 47.5B BG pro-osteogenic effect, hMSCs were seeded in a defined number (1 × 10^4^ cells) onto polystyrene or specimen surface. hMSC were used as representative cells deputed to undergo the self-healing process upon implantation; thanks to their ability to differentiate into bone-like osteoblasts, they were applied to verify the 47.5B BG intrinsic osteoinductivity-inducing hMSC osteogenic differentiation, avoiding the use of any other differentiative biochemicals. After 24 h adhesion, the cells seeded onto polystyrene were cultivated using maintenance medium (DMEM, 10% FBS, 1% antibiotics) or osteogenic differentiative medium (DMEM, 10% FBS supplemented with 0.01 μM dexamethasone, 50 μg/mL ascorbic acid, 10 mM sodium β-glycerophosphate), whereas cells cultivated onto 47.5B BG samples were cultivated only with maintenance medium to avoid any biochemical stimulation. After 3, 6, 9, 12 and 15 days after differentiation induction, the release of the early osteogenic marker alkaline phosphatase (ALP) into the medium was detected by a colorimetric assay (ab83369 kit, from AbCam, UK). At the last time point, the formation of bone-like calcium nodules was histologically verified by the alizarin red staining and images were collected by digital light microscopy; finally, real-time PCR was used to verify the expression of the bone collagen type I (COL I) gene. Briefly, RNA was extracted by TriZol reagent (Sigma), isolated by isopropanol precipitation and reverse-transcribed using a TaqMan kit (from Applied Biosystems, Waltham, MA, USA). For real-time PCR, TaqMan Gene Expression Assays (Applied Biosystems, Thermo Fisher, Waltham, MA, USA) were used on a GeneAmp 7500 Real Time PCR System (Applied Biosystems), using the 18 S rRNA (Applied Biosystems 4310893E) as housekeeping gene. Finally, selected gene expression was normalized towards the starting expression level (intended as the seeding day expression) by the ^ΔΔ^Ct method.

#### 2.2.3. Statistical Analysis

Experiments were performed using three or six replicates. Normal distribution and homoscedasticity were tested with Shapiro–Wilk’s and Levene’s test, respectively. Samples were statistically compared by the SPSS software (v25, IBM, New York, NY, USA) using the one-way ANOVA test and the Tukey’s post hoc analysis. Results were considered as significant for p < 0.05.

### 2.3. Acute Toxicity Tests: Determination of LD_50_ Dose

The study was performed between 2019 and 2020 following the European Communities Council Directives of 24 November 1986, 86/609/EEC and the national guidelines for experimental research on animals. Approval of the Ethical Committee of Uzbekistan under reference no. 9, dated 3 December 2019, was obtained. The animal keeping was consistent with maintenance of experimental biological clinics and sanitary rules. All animals were kept in the same conditions on a normal diet: they were quarantined and acclimatized in vivarium conditions for 14 days at 21 °C, and subjected to 12 h light/dark cycles in accordance with approved norms.

Prior to the determination of the actual LD_50_, a pilot study was conducted with the aim of selecting the dose range for the next stage. In this preliminary study, 30 adult mongrel male mice with initial mass ranging between 20 and 23 g were divided into 5 groups of 6 individuals and subjected to intraperitoneal administration. Briefly, the infusion for injection was prepared as follows: in the first instance, 1 g of glass powder (mean particle size = 16.57 μm; density = 2.64 g/cm^3^; specific surface area = 0.64 m^2^/g [27]) was vigorously mixed in a glass beaker with 9 mL of physiological solution; then, the suspension was stored in a thermostat at 37° C for 24 h and subsequently passed through a filtering paper. Intraperitoneal delivery of the infusion was conducted in the following dose ranges: (500 mg/kg) 0.1 mL/20 g, (1000 mg/kg) 0.2 mL/20 g, (1500 mg/kg) 0.3 mL/20 g, (2000 mg/kg) 0.4 mL/20 g and (2500 mg/kg) 0.5 mL/20 g. After infusion, animals were individually kept in acrylic boxes and observed for the first four hours, 24 h and daily over 14 days for detecting any possible signs of toxicity.

Conversely, for determination of actual LD_50_, 36 mature mongrel male rats with an initial mass of 160–188 g were divided into 6 groups of 6 individuals. The infusion was prepared by suspending glass powder in distilled water containing potato starch (1 g starch/100 g of water). Intragastric route was applied and suspension was delivered at cumulative doses into the stomach through gavage for 0, 12 and 24 h, according to [32,33]. Further treatments were the same as for the intraperitoneal route toxicity assays. The LD_50_ value was calculated by the test-analysis method, i.e., Probit analysis using StatPlus 2009 Professional, 5.8.4. version software [34,35]. At the end of the observation period (14 days), the total number of dead animals was recorded.

### 2.4. In Vivo Biocompatibility Tests

In vivo biocompatibility tests were performed at the Interinstitutional research center, Tashkent Medical Academy, Uzbekistan, on 22 healthy 1-year-old male rabbits from the breed “Chinchilla” weighing 2.8–3.0 kg. The surgeries and animal care carried out following the ethical guidelines and rules of local governmental bodies. The permission for performing in vivo biocompatibility tests was obtained by Ministry of Health of Uzbekistan (the certificate was issued to the Interinstitutional research center, Tashkent Medical Academy, Uzbekistan, under reference no. 3, dated 13 January 2020). Before applying systemic anesthesia, animals were properly kept in individual cages and then identified according to the period and group, as indicated in Table 1.

**Figure 1:**
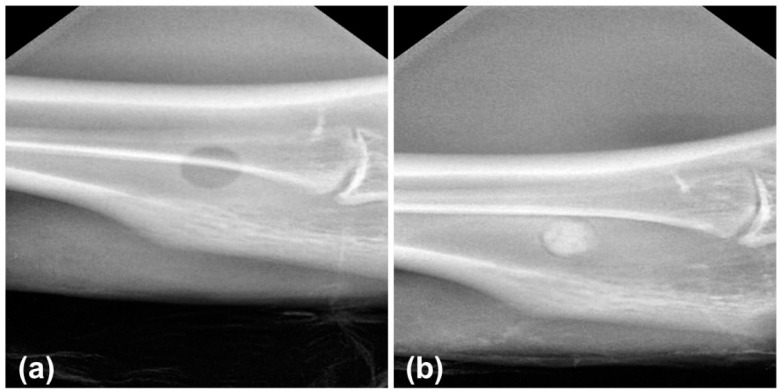
X-ray images of the defect in the animal of the control (**a**) and experimental group (**b**) after surgery.

Prior to surgery, 47.5B silicate glass powder [23,24,27] (mean particle size = 16.57 μm; density = 2.64 g/cm^3^; specific surface area = 0.64 m^2^/g [27]) was sterilized by autoclaving at 180 °C for 2 h and implanted in the femoral diaphysis region of each animal (Figure 1). For additional information about particle characteristics, the reader can refer to Refs. [23,24,27].

**Figure 2:**
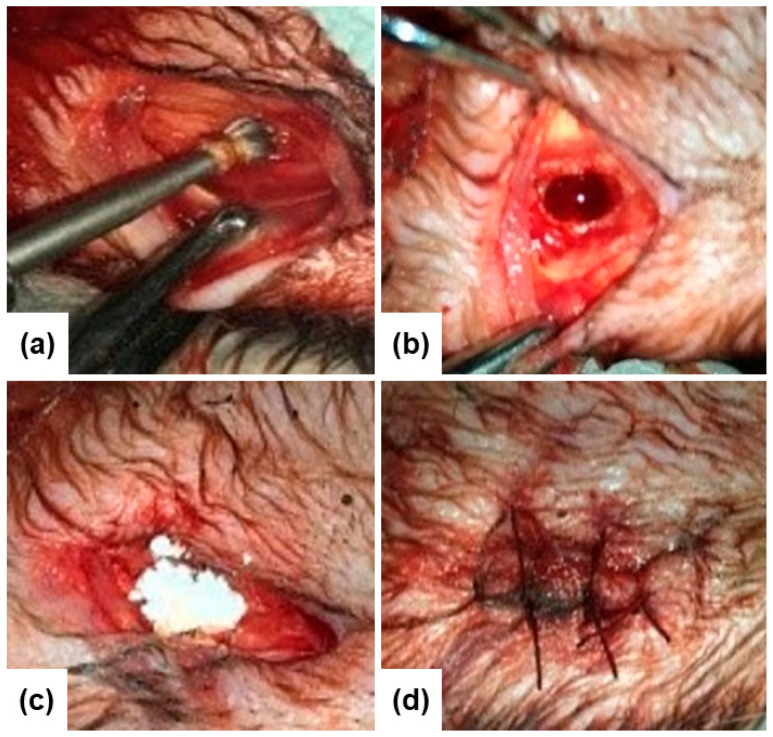
Surgical method to access femoral diaphysis region (**a**), bone defect already created (**b**), bone defect filled with bioactive glass beads (**c**), the incised hole is sewed up (**d**).

Specifically, a drilled hole (diameter = 2 mm; length= 10 mm) was filled out with glass powder and the incised hole was subsequently sewed up (Figure 2). Incision without any implantation was also performed as control.

The X-ray images of the defect in the animal of the control and experimental group after surgery were obtained using DIGIMED Intraoral Digital Dental X-ray Sensor DVS-100.

After surgery, the rabbits from both control and experimental group were returned to their circadian cycle and, at the end of each stage of implantation, were sacrificed by immediate decapitation.

All femurs were fixed in 10% phosphate buffered formalin for 72 h and sent for histopathological analysis to the Faculty of Prosthetic Dentistry, Tashkent State Dental Institute, Uzbekistan. Further, samples were decalcified in 10% formic acid formalin solution for 14 days. The femurs were sectioned parallel to the long axis of the femur through the anteromedial aspect of the defect. The tissue blocks were sectioned and stained with hematoxylin and eosin (H&E) and histopathologically observed by optical microscopy.

The presence and intensity of bone formation were assessed for statistical analysis [36,37] through the following histological scoring scale: (1) no osteogenesis; (2) weak osteogenesis; (3) medium-low osteogenesis; (4) medium-high osteogenesis; (5) good-low osteogenesis; (6) good-high osteogenesis; (7) perfect osteogenesis. Therefore, each slide of histopathological sections was divided into four segments to be observed in detail while the average of the scores of the four quadrants represented the score given to the slide [36].

Wilcoxon–Mann–Whitney test was selected for statistical analysis [36] owing to the fact that 2 groups, i.e., the control and the experimental one, were examined. This type of test involves the calculation of a statistic named U, whose distribution under the null hypothesis is known [38]. The tests were performed with a level of significance of 5%.

## 3. Results and Discussion

The usage of BGs for the clinical management of medium- to critically-sized bone defects cannot prescind from rigorous testing protocols based on in vitro and subsequent in vivo biological models for their validation as bone substitutes, in order to exclude any risks to the patient arising from their use.

### 3.1. In Vitro Cytocompatibility Evaluation

**Figure 3:**
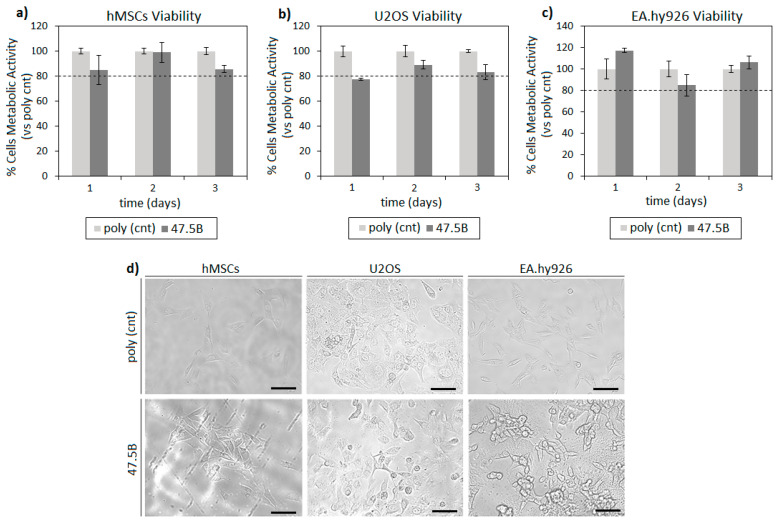
In vitro cytocompatibility of 47.5B specimens. Results were comparable (>80%, *p* > 0.05) between tested BG and polystyrene control (poly cnt) at each time point for all the cell lines (**a**–**c**, bars represent mean ± standard deviation of three replicates). Moreover, optical microscope visual observation (**d**) revealed cells displaying similar morphology, spread and confluence between polystyrene (upper panel) and BG (lower panel). Image bar scale = 100 μm.

In order to test glass cytocompatibility, all the tissue potentially in contact with the implanted material was considered. Accordingly, human mesenchymal stem cells (hMSCs), human bone osteosarcoma cells (U2OS) and human endothelial cells (EA. hy962) were used as representatives for self-healing recruitment, bone and blood vessels, respectively. The main results related to cell viability onto the analyzed substrate are summarized in Figure 3.

The specimens analyzed were cytocompatible towards all the tested cell lines and no significant differences (Figure 3a–c, *p* > 0.05) were observed by comparing the metabolic activity of cells directly cultivated onto the sample surface (47.5B) and the ones cultivated onto polystyrene (gold standard) control. In general, all tested cell lines reported stable metabolic values in function of time that were not significantly reduced in comparison with the polystyrene control: a median value of 91% was observed after 1 day direct cultivation, increasing to 93% after 2 days and to 94% at the last time point of 3 days. Therefore, test specimens can be considered to be safe towards the assayed cell lines. Moreover, the morphology, spread and confluence of hMSCs, U2OS and EA.hy926 cells were visualized by using digital light microscopy after 3 days cultivation (Figure 3d), revealing no differences between cells cultivated onto the polystyrene gold standard (upper panel) and onto BG (lower panel).

### 3.2. In Vitro Pro-Osteogenic Evaluation

**Figure 4:**
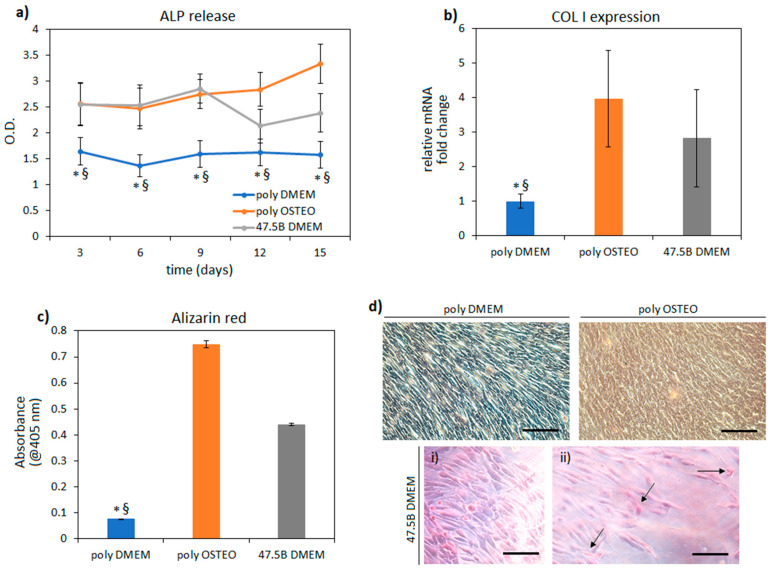
Osteogenic evaluation. During the 15-day cultivation period, the ALP release (**a**) turned out to be significantly higher for cells cultivated onto poly OSTEO and 47.5B BG than for those onto poly DMEM (*p* < 0.05, indicated by * and §). As confirmation, the expression of bone matrix collagen I was overexpressed in both poly OSTEO and 47.5B DMEM (**b**) but not in poly DMEM. Finally, at day 15 the Alizarin red staining quantification (**c**) revealed the presence of calcium deposits for poly OSTEO and 47.5B DMEM; both the groups turned out to be significantly higher than the negative control poly DMEM (*p* < 0.05, indicated by * for poly OSTEO vs. poly DMEM and § for 47.5B BG vs. poly DMEM) as also visually checked by representative images obtained by light microscopy (**d**) showing high cells density (**i**) and frequent calcium deposits ((**ii**), arrows indicate calcium deposits within cells’ aggregates) for cells cultivated onto 47.5B BG specimens. Results represent means ± standard deviation of three replicates; images (representative for salient areas) bar scale =100 μm.

After confirming specimen cytocompatibility, the innovative 47.5B BG was assayed for its potential pro-osteoinductive effect. Therefore, a comparison was performed by relating cells cultivated onto polystyrene using maintenance medium (named poly DMEM) or osteogenic medium (named poly OSTEO) and cells cultivated onto 47.5B specimens using DMEM (named 47.5B DMEM). In this way, the results obtained with the 47.5B specimens were ranked according to a negative (DMEM) and positive (osteogenic medium) control of cells cultivated onto the gold-standard polystyrene to understand whether collagen I (COL I) expression, alkaline phosphatase release and bone-like calcium deposits were promoted or not by the BG chemical composition. Results are summarized in Figure 4.

Considering the hypothesis of these assays, where the performances of 47.5B test specimen were ranked towards negative (poly DMEM) and positive (poly OSTEO) osteogenic controls, it can be concluded that BG investigated in the present study hold pro-osteogenic properties. In fact, the released ALP values (Figure 4a) after 15 days were both significantly higher when poly OSTEO and 47.5B BG groups were compared to poly DMEM controls (*p* < 0.05, indicated by * for poly OSTEO vs. poly DMEM and § for 47.5B BG vs. poly DMEM). ALP is an early osteogenic marker [39], so it can be speculated that the initial differentiative boost towards osteogenesis was similar between the biochemical induction (osteogenic medium) and the chemical one provided by the 47.5B composition. In fact, for those specimens, no other stimulation towards osteoblast proliferation or pro-osteogenic differentiation was provided onto the BG surface as, for example, was previously shown by the authors to promote bone-like matrix formation [39,40]. Similarly, the bone matrix collagen I (COL I, Figure 4b) gene expression revealed that it was overexpressed by the cells belonging to the poly OSTEO and 47.5B B DMEM groups (3.9- and 2.8-fold increase, respectively) in a significant manner as compared to the poly DMEM cells where the expression did not change (<0.05, indicated by * and §) after 15 days cultivation. So, it can be speculated that both the cells belonging to the poly OSTEO and 47.5B DMEM groups successfully differentiated towards the osteogenic phenotype due to biochemical stimulation (poly OSTEO) as well as material chemical composition (47.5B BG). Finally, the Alizarin red staining was used to detect the presence of calcium deposits similar to bone-like nodules after a cultivation period of 15 days (Figure 4c,d). In line with previous results, only the poly OSTEO and 47.5B groups displayed high calcium deposits, thus reporting significant results in comparison with the poly DMEM group (Figure 4c, <0.05, indicated by * and §). Light microscopy images representative for salient areas of cells stained after 15 days (Figure 4d) provided a visual confirmation of the high-density cells (i) as well as of the high percentage of positive cells (ii, stained in red, arrows indicate calcium deposits within the cells cultivated onto 47.5B BG surface) cultivated onto the 47.5B BG in comparison with the positive (poly OSTEO) and negative control (poly DMEM) cells cultivated onto polystyrene. According to these data and the experimental design, it can be concluded that 47.5B B BG samples are pro-osteogenic as their performances are comparable to those observed for the osteogenic medium.

### 3.3. Determination of LD_50_ Dose

When the mice were treated intraperitoneally with the glass powder suspensions, the low doses did not induce any pronounced adverse effect. Constraints in movement and loss of food and water consumption were observed only at relatively high administration doses of the tested material. Nevertheless, no mortality was registered in intraperitoneal administration until the end of the assessments for 14 days. This result aided in the designing of the next step and selection of the dose range for the intragastrical route.

Apart from the symptoms revealed with the intraperitoneal toxicity assay, the rats treated through intragastrical administration additionally demonstrated lethargy, shaking, convulsions, salivation and mortality was registered after 8 days since the beginning of the assay (Table 2).

Using the data from Table 2, the average lethal dose was calculated as LD_50_ = 4522 ± 248 mg/kg. According to the classification of substances by toxicity after intragastric administration, this tested material belongs to the category of slightly toxic substances [17,41].

### 3.4. In Vivo Biocompatibility Tests

No behavioral alterations in animals were observed over the whole implantation period and no adverse immunologic reactions leading to the rejection of the graft occurred. Osteogenesis level was assessed by optical microscopy analysis of tissue blocks, considering the following aspects: (a) the presence of inflammatory infiltrate; (b) the bone marrow vascular niche growth; and (c) the woven bone formation and transformation of woven bone to dense tissue similar to laminar bone. The most striking difference in the bone remodeling process between the experimental and the control groups was observed after 2 and 3 months of implantation. In the present case, after 1 month, no inflammatory infiltration was observed either in the experimental or in the control group. In general, the formation of inflammatory infiltrate is normally observed not later than 2–3 weeks [36,42] since bone damage and the disruption of local blood vessels lead to the exudation of inflammatory cells and hematoma formation.

**Figure 5:**
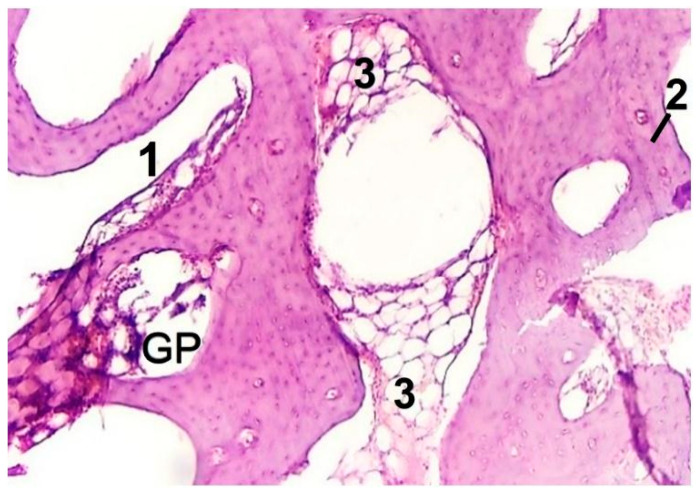
Histopathological sections in cortical area of femur treated with glass powder (experimental group after 2 months, original magnification 10.0×). Legend: GP—residue of bone graft with embedded osteocytes; 1—the bone marrow vascular niche; 2—old bone; 3—fat inclusions to yellow bone marrow.

Bone repair in the form of woven bone appears in intimate contact with the bone graft surface (i.e., left side in Figure 5), which is in accordance with previous observations on other bioactive silicate glass compositions [37,42,43,44]. Moreover, bone graft favors the formation of the bone marrow vascular niche in the cavities of bone. This process is apparently advanced after 2 months (Figure 5, region denoted as 1): bone marrow vascular niche consists of a network of thin-walled sinusoidal vessels whose integrity is maintained and supported by surrounding hematopoietic cells [45]. Additionally, fragmentation of cavities by trabeculae with elements of yellow bone marrow is seen after 2 months (Figure 5, region denoted as 3)**.** Yellow bone marrow is known to contain mesenchymal stem cells and is involved in the storage of fats [45].

**Figure 6:**
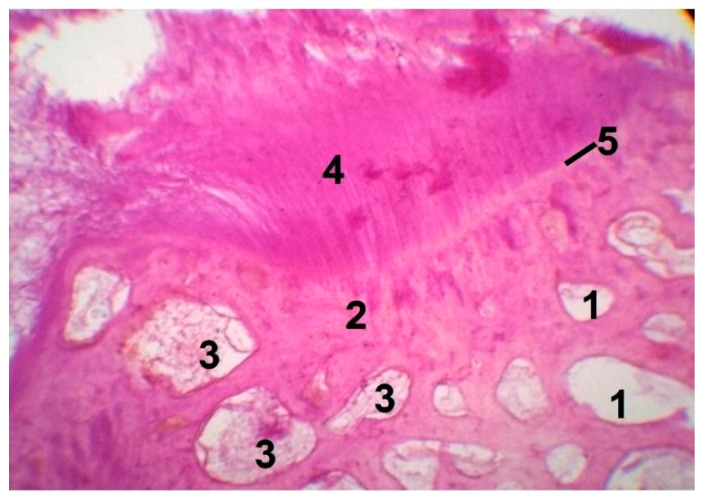
Histopathological sections in cortical area of femur treated with glass powder (experimental group after 3 months, original magnification 10.0×). Legend: 1—the bone marrow vascular niche; 2—old bone; 3—fat inclusions to yellow bone marrow; 4—new bone; 5—the boundaries of bone tissue and bone graft merge (signs of regeneration).

In the experimental group, glass residuals were completely embedded into bone trabeculae after 3 months of implantation, indicating the full resorption of the grafting material in favor of a new homogeneous dense tissue similar to laminar bone (Figure 6), which demonstrated a high level of osteointegration (region denoted as 4) to host bone (region denoted as 2).

The absence of the inflammatory infiltrate in the wound treated with the 47.5B bioactive glass powder shows that this bone graft, although a foreign body, is biocompatible and does not induce an exacerbated inflammatory response. The boundaries of new and old bone tissue merge (Figure 6, region denoted as 5), signifying perfect osteogenesis. In the lower fragment of Figure 6 one can see the osteoid bony plates around the blood vessels. Blood vessel ingrowth is essential to the formation of a soft callus containing fibroblasts and chondroblasts. Furthermore, intramembranous ossification leads to the generation of a bone cuff while endochondral bone formation converts the callus into rigid calcified tissue. Both mineralization and vascular remodeling processes in the callus result in repair of the damaged bone [46,47,48].

**Figure 7:**
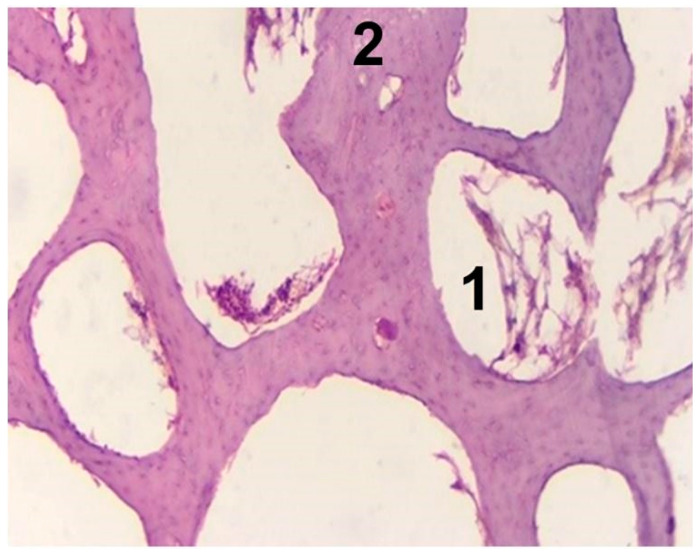
Histopathological sections in cortical area of femur (control group after 3 months, original magnification 10.0×). Legend: 1—the bone marrow vascular niche; 2—old bone.

In general, the experimental group demonstrated different steps of bone remodeling, especially in the areas close to the implantation site. Unlike the experimental group, the control group defect even after 3 months was still filled with loose connective tissue and demonstrated incomplete compaction (Figure 7, Table 3).

Wilcoxon–Mann–Whitney test used to perform statistical analysis (data for calculation are shown in Table 3) showed that U calculated ≤ U critical always, i.e., calculated U values were either equal to or lower than critical tabulated U value. Therefore, it was suggested that there is a statistically significant difference between intensity of bone formation score in the control and the experimental groups at all 3 tested periods of implantation (Table 3). In other words, the “osteogenetic effect” is statistically significant, it is real and it is not due to chance.

Fracture healing is a complex biological process which is still not fully understood. The process includes first the development of an acute inflammatory response, followed by the recruitment of mesenchymal stem cells (MSCs), the generation of cartilaginous bony callus, the mineralization of the extracellular matrix (ECM), revascularization/angiogenetic processes, resorption of cartilaginous callus and finally bone remodeling [49,50]. It was proposed [50] that new bone formation begins in the bone marrow, gradually progressing towards the periphery of the defect. Bone regeneration is not uniform over the whole volume of the defect and, in general, the healing rate of the core is slower compared to the surrounding areas. When a drilling defect is created in the femoral diaphysis of young rabbits, as in the present case, blood fills the defect and fibrinous arcades form within the blood clot across the defect from wall to wall. Then, MSCs are arranged parallel to the fibrinous arcade, in conjunction with vascularization. Furthermore, MSCs differentiate to mesenchymal osteoblasts, with increasing woven tissue synthesis, and eventually to surface osteoblasts synthesizing new lamellar on woven bone tissue. Over time, the conversion of woven bone into organized lamellar may occur, thus allowing the restoration of the canal and the physiological properties of bone [50].

The results reported in the present study further support the capability of bioactive glasses—in this specific case the 47.5B composition—to act as an inherently osteoinductive biomaterial that, after being implanted, can stimulate bone regeneration *per se*. This does not occur for other implantable biomaterials, such as hydroxyapatite [51], that are used for bone repair although being only osteoconductive, and is a significant advantage over other clinical approaches, such as the administration of growth factors whose efficacy and safety are still debated [52].

## 4. Conclusions

In the present study, the in vitro and in vivo biological responses of a bioactive silicate glass in the SiO_2_-Na_2_O-K_2_O-MgO-CaO-P_2_O_5_ system were investigated. In vitro tests revealed good interaction of the glass surface with all the cell types analyzed, showing the same level of cytocompatibility as the positive control material. Moreover, a clear intrinsic pro-osteogenic effect mainly related to the chemical composition of the material was observed, leading to the differentiation of mesenchymal stem cells.

In vivo tests demonstrated the existence of a statistically significant difference between the intensity of bone formation score in the control and the experimental groups at all three tested periods of implantation. Thus, 47.5B glass particulate elicited an osteogenetic effect and was prone to almost completely resorb within 3 months post-implantation. New homogeneous dense tissue similar to laminar bone formed after 3 months at the expense of bone graft.

The high level of osteointegration observed, together with the absence of a severe inflammatory response, confirmed the potential of 47.5B glass to be used in osteostimulatory bone healing applications.

## Figures and Tables

**Table 1:** Design of the implantation procedure.

Observation Stage	Glass Powder (Experimental)	Empty Hole (Control)
1 month	3 animals per group with numbering in the range No.1–3	3 animals per group with numbering in the range No.4–6
2 months	4 animals per group with numbering in the range No.7–10	4 animals per group with numbering in the range No.11–14
3 months	4 animals per group with numbering in the range No.15–18	4 animals per group with numbering in the range No.19–22

**Table 2:** Mortality resulting from the intragastric administration route.

Dose (mg/kg)	Number of Dead Rats/Total Number of Rats
4000	0/6
4100	1/6
4250	2/6
4500	4/6
4750	4/6
5000	6/6

**Table 3:** Bone formation score for experimental and control groups after 2 and 3 months of implantation according to histological scoring scale.

Observation Stage	Glass Powder (Experimental Group)	Empty Hall (Control)
2 months	No.7/ score 4 No.8/ score 4 No.9/ score 4 No.10/ score 5	No.11/ score 2 No.12/ score 3 No.13/ score 3 No.14/ score 3
3 months	No.15/ score 5 No.16/ score 5 No.17/ score 6 No.18/ score 7	No.19/ score 3 No.20/ score 4 No.21/ score 4 No.22/ score 3

## Data Availability

Data available in the study.
